# Accuracy of 12 IOL power calculation formulas in highly myopic eyes

**DOI:** 10.1007/s10792-025-03608-0

**Published:** 2025-06-25

**Authors:** Magí Vilaltella, Pau Cid-Bertomeu, Tonet Serés-Noriega, Valentín Huerva

**Affiliations:** 1 Department of Medicine and Surgery, Faculty of Medicine, University of Lleida, Carrer de Montserrat Roig, 2, 25008 Lleida, Spain; 2 Department of Ophthalmology, University Hospital Arnau de Vilanova, Lleida, Spain; 3 Department of Endocrinology and Nutrition, Centro Médico Milenium, Saragossa, Spain

**Keywords:** Intraocular lens power calculation formulas, Long eyes, Highly myopic eyes, Cataract surgery

## Abstract

**Purpose:**

To assess and compare the accuracy of 12 intraocular lens (IOL) power calculation formulas after cataract phacoemulsification surgery in eyes with an axial length (AL) greater than or equal to 26.00 mm (≥ 26.00 mm).

**Methods:**

A retrospective case series study including 72 eyes with an AL ≥ 26.00 mm that underwent uneventful cataract surgery. Twelve IOL power calculation formulas were evaluated: SRK/T, Holladay 1, Haigis, Holladay 2, Barrett Universal II, Kane, EVO, Pearl-DGS, Hill RBF 3.0, SRK/T and Holladay 1 with the modified W-K AL adjustment, and Holladay 2 with the non-linear W-K AL adjustment. The median absolute error (MedAE), mean absolute error (MAE) and the percentage of eyes within specific prediction error thresholds were calculated and compared across formulas after adjusting the mean error to zero.

**Results:**

Holladay 1_modified W-K_ obtained the lowest MedAE (0.215 DP), followed by Kane (0.233 DP), Barrett (0.246 DP), Pearl and EVO (0.252 DP). Barrett, Kane and EVO yielded significantly lower MedAEs compared to SRK/T (*p* < 0.05); and Holladay 1_modified W-K_ yielded significantly lower MedAEs compared to SRK/T_modified W-K_ (*p* < 0.05). Holladay 1_modified W-K_ achieved the lowest MAE (0.314 DP), followed very closely by Barrett (0.317 DP), and subsequently by Pearl (0.324 DP), Kane (0.329 DP) and EVO (0.331 DP).

**Conclusion:**

Our study reflects a superior accuracy of the Holladay 1_modified W-K_ formula, along with the Kane, Barrett, EVO and Pearl-DGS formulas in predicting refractive outcomes for eyes with an AL greater than 26.00 mm undergoing cataract phacoemulsification surgery.

**Supplementary Information:**

The online version contains supplementary material available at 10.1007/s10792-025-03608-0.

## Introduction

Refractive accuracy is one of the pillars of successful cataract surgery [1]. The accuracy of intraocular lens (IOL) power implantation is determined not only by the precision of preoperative biometric measurements—such as axial length (AL), which is responsible for around 36% of postoperative refractive errors—but also by the choice of the IOL power calculation formula [2, 3]. There currently are many different intraocular lens (IOL) power calculation formulas to achieve this goal. These formulas can be classified as either vergence, ray tracing, artificial intelligence (AI) or a combination approach [4]. Most of them are useful for eyes with an axial length (AL) between 22 and 26 mm. However, formulas appear to be less accurate outside these boundaries of AL.

Specifically for long eyes, the third-generation formulas (SRK/T, Holladay 1 and Hoffer Q) tend to underestimate IOL power, causing hyperopic outcomes [5]. For this reason, many surgeons tend to aim for a myopic postoperative refractive target of − 1.00 or even − 2.00 diopters (DP) when selecting the IOL power for cataract surgery [6].

*Wang *et al. [6] proposed a method for optimizing AL for the Holladay 1, SRK/T, Haigis and Hoffer Q formulas, that significantly reduced the percentage of long eyes with a hyperopic outcome. However, the authors later modified the original AL optimization formulas for Holladay 1 and SRK/T (excluding Hoffer Q and Haigis) by using ULIB lens constants (instead of manufacturers’) and manifest refraction converted to 6 m, in order to make them less aggressive and improve refractive outcomes [7]. The Holladay 1 and SRK/T formulas combined with original and modified W-K (Wang-Kock) AL adjustments have shown similar accuracy compared to fourth-generation formulas for highly myopic eyes [8, 9]. Similarly, Wang et al. [10, 11] published two AL optimization equations (linear and non-linear) for the Holladay 2 formula, which have been less evaluated in the literature.

The newest formulas such as EVO, Kane and Hill-RBF 3.0, along with Barrett Universal II (BU II), have also proven to be more accurate in predicting the post-operative refractive error than the older formulas for myopic eyes [4, 12–18]. Among these, two formulas seem to obtain the best results in recently published studies about IOL power calculation for long eyes: Kane and EVO [12, 14, 19]. The Kane formula was developed in 2017 by Dr Jack Kane and combines theoretical optics, regression and artificial intelligence (AI) based on a huge database to make its predictions [14]. The EVO 2.0 formula was developed by Dr Tun Kuan Yeo and is based on the theory of emmetropization and the thick lens formula [20].

Pearl-DGS is a thick lens and AI-based formula, launched in 2017 by Debellemanière, Gatinel and Saad [21]. The performance of this formula has been reported to be comparable to the other formulas included in the ESCRS calculator (Barrett, Kane, EVO, Hill-RBF and Hoffer QST) in the long AL subgroup and across the entire AL range [22]. However, further studies may be required to assess the accuracy of Pearl-DGS in myopic eyes.

The objective of this study is to assess and compare the accuracy of 12 IOL calculation formulas in eyes with an AL greater than or equal to 26.00 mm (≥ 26.00 mm).

## Methods

This is a retrospective case-series study. Ethical approval was obtained from the Ethics Committee of Hospital Universitari Arnau de Vilanova de Lleida (HUAV, Spain). Patients’ data were collected from the electronic medical record of our centre (SAP software; Systems, Applications, Products in Data Processing). Analyses of the outcomes were conducted following the recommendations established by *Wang *et al. [23] for IOL power calculation formulae studies.

All cataract surgeries performed at HUAV from May 2014 to February 2021 were reviewed. All eyes with an AL greater than or equal to 26.00 mm that underwent sutureless phacoemulsification cataract surgery with in-the-bag IOL implantation were included. Preoperative biometry was measured using the IOL Master 500 biometer (IOL Master V 3.1, Zeiss) and postoperative subjective refraction was obtained by an optometrist at our centre at least 1 month after the surgical procedure. In all cases, one of the following IOL models was implanted: SN60WF, MN60MA (Alcon Laboratories, Inc.), or EyeCee®One (Bausch + Lomb, Inc.). The SN60WF and EyeCee One IOLs have a monofocal, one-piece, hydrophobic acrylic design with aspheric optics, whereas the MN60MA has a three-piece, monofocal, hydrophobic acrylic design with a spherical optic [F]. Exclusion criteria were as follows: 1) preoperative corneal astigmatism higher than 3.50 DP, 2) previous intraocular or corneal refractive surgery, 3) previous corneal disease, 4) postoperative best corrected visual acuity (BCVA) lower than 20/40, 5) complicated cataract surgery, 6) postoperative complications or 7) incomplete documentation.

**Fig. 1:**
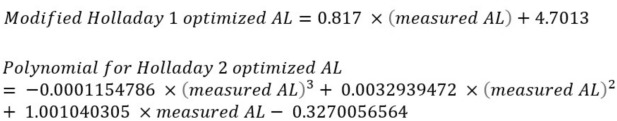
Axial length adjustment formulas

Twelve different IOL power calculation formulas were evaluated. SRK/T, SRK/T with the modified W-K AL adjustment (SRK/T_modified W-K_), Haigis, Holladay 1, Holladay 1 combined with the modified W-K AL adjustment (Holladay 1_modified W-K_), Holladay 2 and Holladay 2 combined with the non-linear polynomial W-K AL adjustment (Holladay 2_NP W-K_) were calculated using the software included in the IOL Master 700 (Zeiss). The W-K AL adjustments were carried out as reported by *Wang *et al. [7, 10, 11] before introducing the adjusted AL values into the IOL Master 700 multiformula calculator. The AL adjustment formulas [7, 10, 11] applied are detailed in Fig. 1. Hill RBF 3.0, Kane, EVO, Barrett and Pearl-DGS were calculated using their respective websites [A-E]. The following biometric variables were used for the IOL power calculation: anterior chamber depth (ACD), AL, K1 (flat keratometry) and K2 (steep keratometry). No values of lens thickness (LT) or central corneal thickness (CCT) were employed. Lens constants used for SN60WF were those that appear as “adopted from ULIB” on the IOLcon [F] database. For the MN60MA and the EyeCee®One lens we used the A constant provided by the manufacturer. The Holladay 2 ACD constant for SN60WF and EyeCee®One, and the remaining constants for MN60MA (a1, a2 and a3 for Haigis, SF for Holladay 1 and ACD for Holladay 2) were directly obtained from the conversion made by the IOL Master 700 software after introducing the A constant for the SRK/T formula.

Refractive prediction error (RPE) was calculated as the actual postoperative subjective refraction minus the predicted refraction. The mean error (ME(SD)) and the percentage of hyperopic refractive errors were calculated for each formula. In order to eliminate the systematic error derived from the use of a non-optimized constant, we zeroed out the mean refractive prediction error for each formula by modifying the RPE for each eye up or down by an amount equal to the ME, as described by *Wang *et al. [23]. The following parameters were calculated after zeroing out the mean RPE: 1) median absolute prediction error (MedAE [IQR]), 2) mean absolute prediction error (MAE (SD)) and 3) percentage of eyes within a prediction error of ± 0.25 DP, ± 0.50 DP, ± 0.75 DP, and ± 1 DP.

Descriptive and inferential statistics were conducted using Excel (Office 365, Microsoft Corp.) and STATA (v.17, StataCorp LLC), respectively. Data distribution was assessed for normality using the Shapiro-Wilks test and by visualization of the P-P plots. Depending on whether the refractive errors followed a normal distribution, either the one-sample t-test or the one-sample Wilcoxon signed rank-test were used to evaluate whether the ME was significantly different from 0. Differences in the absolute prediction errors between formulas were evaluated using the Friedman test and, in the event of a significant outcome, post-hoc analysis was carried out using the paired samples Wilcoxon test. Differences in the percentage of eyes with hyperopic refractive outcomes and in the percentage of eyes within specific prediction error ranges among formulas were assessed using the Cochrane Q test, and in case of a significant result, post-hoc analysis was performed using the McNemar test. The Bonferroni correction was applied for multiple comparisons. Regression analysis using generalized estimating equations (GEE) was performed to explore the association between biometric variables (AL, ACD, and mean-K) and the RPE for each formula. This method has been employed in previous studies to account for the correlation between pairs of eyes [24, 25]. Statistical significance was set at a p-value of less than 0.05 (*P* < 0.05).

Sample size calculation was performed by analysis of variance (ANOVA) for repeated measures. To achieve a power of 80% with an alpha error of 5% (corresponding to a 95% confidence level), 71 eyes were required.

## Results

Seventy-two eyes of fifty-three patients were recruited for the study after applying the inclusion and exclusion criteria. Demographic and biometric data are presented in Table 1. IOL constants used are listed in Table 2.

**Table 1:** Demographic and biometric data

	Count (% of Total)
Female sex	38
IOL type	
SN60WF Acrysof	55
MN60MA (+ D) Acrysof	6
MN60MA (-D) Acrysof	2
B&L SZ-1 EyeCee One	9
	Mean (SD)
Age	66 (10.76)
IOL power	10.53 (4.10)
Axial Length	27.37 (1.53)
Average Keratometry	43.48 (1.48)
Anterior Chamber Depth^a^	3.46 (0.33)

IOL, intraocular lens

^a^Anterior chamber depth measured from corneal epithelium to lens

**Table 2:** IOL constants used

Formula	Constants	SN60WF	MN60MA (+ and − D)	EyeCee One
SRK/T, Hill RBF 3.0, Kane, EVO, Barrett Universal II, Pearl-DGS	A	119^a^	118.9^b^	119.7^b^
Holladay 1	sF	1.84^a^	1.67^c^	2.13^b^
Holladay 2	ACD	5.601^c^	5.491^c^	5.900^c^
Haigis	a_0_, a_1_, a_2_	− 0.769, 0.234, 0.217^a^	1.243, 0.400, 0.100^c^	1.675, 0.400, 0.100^b^

IOL, intraocular lens; sF, surgeon factor; ACD, anterior chamber depth

^a^Optimized constant from IOL Con database (adopted from ULIB)

^b^Manufacturer-provided constant

^c^Constant obtained from the conversion performed by the IOL Master 700 using the A-constant

### Refractive errors using ULIB constants

Outcomes of ME(SD) and percentages of hyperopic results for each formula before eliminating the systematic error are illustrated in Table 3.

**Table 3:** Refractive outcomes using ULIB constants

Formula	ME(SD), (D)	Range (D)	% of hyperopic prediction errors
Hill RBF 3.0	0.153 (± 0.450)^a^	− 0.95, + 1.36	67^c^^,d^
Kane	0.180 (± 0.429)	− 0.84, + 1.48	71^c^
EVO	0.227 (± 0.426)	− 0.75, 1.53	74^c^
Barrett Universal II	0.176 (± 0.405)	− 0.94, + 1	71^c^
Pearl-DGS	0.135 (± 0.427)^a^	− 0.93, + 1.39	68^c^^,d^
Holladay 2	0.477 (± 0.502)	− 0.47, + 2.06	85
Holladay 1	0.633 (± 0.511)	− 0.34, + 2.18	92
Haigis	0.308 (± 0.473)	− 0.70, + 1.90	76
SRK/T	0.321 (± 0.524)	− 0.75, 1.69	76
Holladay 2_NP W-K_	− 0.082 (± 0.451)^a^	− 1.30, + 1.16	43^b^
Holladay 1_modified W-K_	0.086 (± 0.428)^a^	− 0.97, + 1.36	64^c^^,d^
SRK/T_modified W-K_	0.079 (± 0.475)^a^	− 1.04, + 1.20	65^c^^,d^

ME, mean error; SD, standard deviation; D, dioptre

^a^ME significantly different from 0 (*p* < 0.05)

^b^*p* < 0.05 Holladay 2_NP W-K_ vs all other formulas

^c^*p* < 0.05 Hill, Barrett, Kane, EVO, Pearl, SRK/T_modified W-K_ and Holladay 1_modified W-K_ vs Holladay 1

^d^*p* < 0.05 Hill, Pearl, SRK/T_modified W-K_ and Holladay 1_modified W-K_ vs Holladay 2

The ME of Hill RBF 3.0, Pearl-DGS, SRK/T_modified W-K_, Holladay 1_modified W-K_ and Holladay 2_NP W-K_ was not significantly different from 0 (*p* > 0.05). BU II, Kane, EVO, SRK/T, Haigis, Holladay 1 and Holladay 2 induced hyperopic MEs (*p* < 0.05). Detailed results of the statistical analysis are summarized in Supplementary Table 1 (Online Resource 1).

The formulas that resulted in a significantly lower percentage of hyperopic refractive errors were the following: 1) Holladay 2_NP W-K_ compared to all other formulas (43% vs 63–91%); 2) Hill, Barrett, Kane, EVO, Pearl, SRK/T_modified W-K_ and Holladay 1_modified W-K_ compared to Holladay 1 (63–73% vs 91%) and 3) Hill, Pearl, SRK/T_modified W-K_ and Holladay 1_modified W-K_ compared to Holladay 2 (63–68% vs 84%) (*p* < 0.05). Detailed results of the statistical analysis are summarized in Supplementary Table 2 (Online Resource 1).

The biometric variables that significantly contributed to the RPE for each formula are presented in Table 4. Mean K demonstrated a negative correlation with the RPE for Holladay 1, Holladay 1_modified W-K_, SRKT, SRK/T_modified W-K_, Barrett, EVO, Hill and Kane. AL showed a positive correlation with the RPE for Holladay 2 and Haigis, and a negative correlation with the RPE for Holladay 2_NP W-K_ (*p* < 0.05). Detailed results of the statistical analyses performed to elaborate the GEE are summarized in Supplementary Table 3 (Online Resource 1).

**Table 4:** Biometric parameters contributing to the refractive prediction error

Formula	Refractive prediction error GEE
Hill RBF 3.0	− 4.700 − 0.105 × mean K
Barrett Universal II	3.327 − 0.072 × mean K
EVO	3.328 − 0.071 × mean K
Kane	4.153 − 0.091 × mean K
Holladay 2	− 4.805 + 0.193 × AL
Holladay 1	− 1.132 + 0.179 × AL − 0.072 × mean K
Haigis	− 2.962 + 0.119 × AL
SRK/T	2.229 + 0.151 × AL − 0.139 × mean K
Holladay 2_NP W-K_	3.003 − 0.113 × AL
Holladay 1_modified W-K_	3.243 − 0.072 × mean K
SRK/T_modified W-K_	6.123 − 0.139 × mean K

GEE, generalized estimating equation; mean K, mean keratometry; AL, axial length

### Refractive errors after eliminating systematic errors

**Fig. 2:**
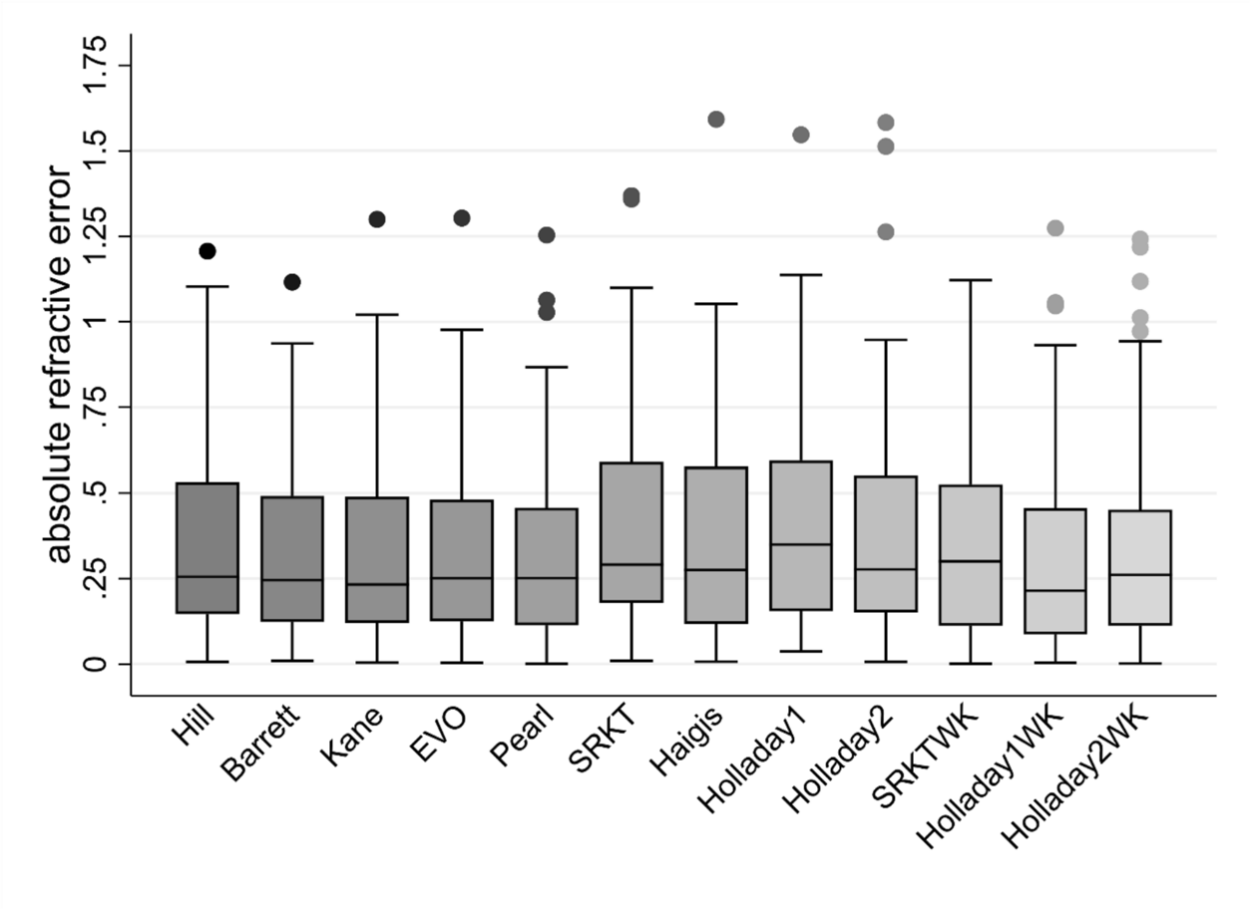
MedAE after eliminating systematic errors

Outcomes of MedAE [IQR], MAE (SD) and percentage of eyes within specific prediction errors (± 0.25 DP, ± 0.50 DP, ± 0.75 DP, ± 1 DP) after adjusting the ME to zero are summarized in Table 5. A boxplot showing the distribution of absolute refractive errors around the MedAE for each formula is presented in Fig. 2.

**Table 5:** Refractive outcomes after eliminating systematic errors

Formula	ME (SD), (D)	Range (D)	MedAE [IQR], (D)	MAE ± SD	% ± 0.25 D	% ± 0.50 D	% ± 0.75 D	% ± 1 D
Holladay 1_modified W-K_	0.000 (± 0.428)	1.274, − 1.056	0.215 [0.367]^a^	0.314 ± 0.289	56	78	92	96
Kane	0.000 (± 0.429)	1.300, − 1.020	0.233 [0.365]^b^	0.329 ± 0.274	54	76	92	97
BU II	0.000 (± 0.405)	0.824, − 1.116	0.246 [0.364]^b^	0.317 ± 0.249	51	76	93	99
EVO	0.000 (± 0.426)	1.300, − 1.020	0.252 [0.354]^b^	0.331 ± 0.266	50	76	93	99
Pearl-DGS	0.000 (± 0.427)	1.254, − 1.063	0.252 [0.337]	0.324 ± 0.276	50	78	92	96
Hill RBF 3.0	0.000 (± 0.450)	1.207, − 1.103	0.255 [0.383]	0.349 ± 0.282	49	69	90	97
Holladay 2_NP W-K_	0.000 (± 0.451)	1.242, − 1.218	0.261 [0.334]	0.341 ± 0.292	49	79	90	94
Haigis	0.000 (± 0.473)	1.592, − 1.008	0.275 [0.447]	0.358 ± 0.307	47	69	93	96
Holladay 2	0.000 (± 0.502)	1.547, − 0.968	0.277 [0.390]	0.378 ± 0.328	44	71	90	96
SRK/T	0.000 (± 0.524)	1.369, − 1.071	0.291 [0.401]	0.414 ± 0.317	46	63	83	94
SRK/T_modified W-K_	0.000 (± 0.475)	1.121, − 1.119	0.300 [0.404]	0.360 ± 0.307	46	74	85	96
Holladay 1	0.000 (± 0.511)	1.547, − 0.968	0.350 [0.429]	0.408 ± 0.303	38	68	86	96

MedAE, median absolute error; ME, mean error; MAE, mean absolute error; SD, standard deviation; D, dioptre; BU II, Barrett Universal II; IQR, interquartilic range

^a^*p* < 0.05 Holladay 1_modified W-K_ vs SRK/T_modified W-K_

^b^*p* < 0.05 Barrett Universal II, Kane and EVO vs SRK/T

Overall, Holladay 1_modified W-K_ obtained the lowest MedAE (0.215 DP), followed by Kane (0.233 DP), Barrett (0.246 DP), Pearl, and EVO (0.252 DP). Barrett, Kane and EVO yielded significantly lower MedAEs compared to SRK/T (*p* < 0.05); and Holladay 1_modified W-K_ yielded significantly lower MedAEs compared to SRK/T_modified W-K_ (*p* < 0.05). Statistics of significant results are summarized in Supplementary Table 4 (Online Resource 1). No other statistically significant differences in terms of MedAE were found between formulas.

Regarding the MAE outcomes, Holladay 1_modified W-K_ produced the lowest value (0.314 DP), followed very closely by Barrett (0.317 DP), and subsequently by Pearl (0.324 DP), Kane (0.329 DP) and EVO (0.331 DP).

Holladay 1_modified W-K_ had the highest percentage of refractive errors within the range below 0.25 DP (55%), followed by Kane (54.17%) and Barrett (51.39%). Holladay 2_NP W-K_ had the highest percentage of refractive errors within the range below 0.50 DP (79.17%), followed by Holladay 1_modified W-K_ and Pearl (77.78%), and subsequently by Barrett, Kane and EVO (76.39%). No significant differences were observed in the percentage of eyes within specific refractive error thresholds (0.25 DP, 0.50 DP, 0.75 DP, 1 DP) among formulas (*p* < 0.05).

## Discussion

In our study, Holladay 1_modified W-K_ yielded the best results in terms of MedAE, followed closely by Kane; and subsequently by Barrett, Pearl and EVO. Statistically significant differences were observed when comparing the MedAE of three new-generation formulas (Barrett, Kane and EVO) with the older SRK/T. Additionally, we found significant differences in the MedAE between Holladay 1_modified W-K_ and SRK/T_modified W-K_.

In terms of MAE, the Holladay 1_modified W-K_ and the Barrett formulas produced the most accurate results, followed again by other new-generation formulas (Pearl, Kane and EVO). There were no significant differences in the percentage of eyes within specific prediction error ranges among formulas. However, it is important to highlight that the Holladay 1_modified W-K_ had the highest percentage of refractive errors within the range below 0.25 DP, followed by Kane and Barrett. Additionally, Holladay 2_NP W-K_ had the highest percentage of refractive errors within the range below 0.50 DP, followed by Holladay 1_modified W-K_.

Our study is consistent with previous literature, which indicates a greater accuracy of the new-generation formulas and the Wang-Koch AL adjustment formulas for IOL power calculation in eyes with axial myopia. However, the question of which new-generation formula or WK AL adjustment formula performs better varies across studies. In a study involving 370 long eyes, Cheng et al. [26] reported the superiority of the Holladay 1-WK (both original and modified versions) and Kane formulas over the EVO and SRK/T-WK (original and modified) formulas, with statistically significant differences in the MedAE. In that study [26], the Kane and Holladay 1-WK formulas also achieved a lower MedAE compared to Hill RBF 2.0 and BU II. In another investigation including 164 myopic eyes, Zhang et al. [8] observed that the Holladay 1 formula, with both the first linear and nonlinear versions of the WK AL adjustment, and the EVO formula showed the lowest MAE and MedAE across all formulas; while Barrett and SRK/T with original AL adjustment displayed a slightly but not significantly lower accuracy. However, the modified WK AL adjustment for the Holladay 1 formula resulted in a significantly higher MedAE compared to the original version. In a study involving 136 long eyes, Liu et al. [24] observed that the Barrett formula produced the lowest MAE and MedAE values, followed by Hill RBF 2.0, Holladay_original W-K_ and Holladay 1_modified W-K_. In the mentioned study [24], Barrett obtained significantly lower MAEs than did the Holladay 1, SRK/T and both the original and modified SRK/T WK AL adjustment formulas; and the Hill RBF 2.0 and the Holladay_original W-K_ obtained significantly lower MAEs than did the SRK/T formula. Moreover, Barrett and Hill produced a significantly higher percentage of eyes within a 0.5 DP prediction error compared to Holladay 1, SRK/T and SRK/T_modified W-K_. In a subgroup analysis of eyes with an AL greater than or equal to 26.00 mm, Savini et al. [19] reported that the EVO formula yielded the lowest MAE and MedAE, followed by Kane and Barrett. The only WK AL adjustment formula employed was for the Holladay 2 formula, which produced substantially worse outcomes compared to EVO and Kane. Darcy et al. [14], in a subgroup analysis of 637 long eyes, found that the Kane formula had a significantly lower MAE compared to the Hill 2.0, Barrett, Holladay 2, Olsen, Haigis, and third-generation formulas. Additionally, Barrett also had a significantly lower MAE compared to all the formulas mentioned, except for Kane. A retrospective study comparing the Kane, Barrett, EVO, Haigis and SRK/T formulas in 175 eyes with high axial myopia reported that, in the subgroup of eyes with an AL between 26 and 28 mm, the EVO formula demonstrated significantly higher accuracy [12]. The study concluded that, overall, the Kane and EVO formulas achieved better results compared to the other formulas [12]. Finally, a recent investigation comparing seven AI-based formulas in 48 eyes with extremely long axial length (AL > 30 mm) found that the Hill-RBF 3.0 formula yielded the lowest root mean square absolute error (RMSAE), with statistically significant differences compared to the Karmona formula [27]. Meanwhile, the lowest MedAE was achieved by the Hoffer QST formula, followed by Kane and Pearl-DGS [27].

Pearl-DGS is a thick lens and AI-based formula, launched in 2017 by Debellemanière, Gatinel and Saad [21]. The performance of this formula has not been extensively studied in long eyes; therefore, we decided to include it in our study. We found that, in terms of MAE and MedAE, the Pearl-DGS formula achieved an accuracy comparable to other new-generation formulas (Kane, EVO, Barrett); however, no statistically significant differences were observed between Pearl-DGS and any of the other formulas included in our work. In a subgroup analysis of long eyes within a study comparing the formulas included in the ESCRS calculator [22], the authors observed that the Pearl-DGS formula achieved similar results to the other formulas in terms of MAE and MedAE. In the same study [22], the Kane formula demonstrated a significantly lower MAE compared to Barrett, Hoffer QST and EVO; and the Kane and Hill 3.0 formulas demonstrated a significantly lower MedAE compared to Hoffer QST, in the long-eye subgroup analysis.

Although the differences in refractive prediction error observed between formulas in our study were modest (approximately 0.10 D), they are consistent with those commonly reported in comparative studies of IOL power calculation formulas. We believe these differences are clinically relevant, as formulas that consistently perform better in controlled studies often yield noticeably superior outcomes in real-world clinical settings. Even small improvements in prediction accuracy can contribute to higher rates of emmetropia and patient satisfaction when applied across larger and more diverse patient populations. Therefore, we believe these findings have practical implications for surgical planning and formula selection.

As shown in the multiple regression analysis evaluating the association between biometric variables and refractive prediction error for each formula in our study (Table 4), certain formulas appear to be influenced by variability in specific biometric parameters. Consequently, differences in the biometric profile of patient populations across studies may contribute to the discrepancies in formula performance reported in the literature. When comparing the mean values of keratometry (K), anterior chamber depth (ACD), and axial length (AL) in our sample to those reported in other studies involving eyes with AL > 26.00 mm, we observe that our mean K and ACD values are very similar to those in previous reports. However, with respect to mean AL, while some studies report comparable values [8, 28], others include populations with considerably longer eyes (mean AL ranging from 28.78 to 29.63 mm) [9, 24, 26, 29–31]. This variation should be considered when interpreting the generalizability of our findings.

We decided to include several earlier IOL power calculation formulas in our investigation. SRK/T, Holladay 1, Haigis, and Holladay 2 are vergence formulas based on thin-lens optics [21]. All of them were included in the study due to their widespread use over the past decades, their demonstrated accuracy in IOL power calculation, and their continued use in clinical practice worldwide. We believe it is important to compare them with newer formulas to determine whether recent developments truly offer improved refractive prediction.

A limitation of our study is the relatively small number of eyes included (72), especially in relation to the high number of formulas analysed (12), which restricts the ability to detect statistically significant differences. In addition to its impact on statistical power, this also limits the generalizability of the results, which would likely have been greater with a more diverse and representative sample. The difficulty of retrospectively identifying long eyes that meet the inclusion criteria makes it challenging to achieve a large sample size. Nevertheless, the strict inclusion and exclusion criteria applied enhance the reliability and reproducibility of our study. Moreover, the primary aim of this study is not solely to identify statistical differences between formulas, but also to serve an exploratory purpose by generating hypotheses that can be tested in future studies.

Similarly, both eyes of some patients were included in the analysis, which may have influenced the statistical results due to the potential correlation between data from bilateral eyes [32]. To account for the correlation between pairs of eyes, we performed regression analysis with generalized estimating equations to explore the association between biometric variables and the RPE for each formula, as reported in previous studies [24, 25]. Also, we employed three different IOL models, which may introduce variability and serve as a potential source of error [32].

Recently, a new predictive error metric known as the root mean square absolute error (RMSAE) has been proposed for evaluating the performance of IOL power calculation formulas [33]. We did not include this measure in our analysis, as we employed more established metrics such as the MAE, MedAE, and the percentage of eyes within specific prediction error thresholds. However, the exclusion of RMSAE may be considered a limitation, as it has been reported to be a reliable indicator and has been utilized in recent studies [27, 34, 35].

The absence of LT and CCT represents another limitation, as it may affect the accuracy of some formulas employed in our investigation. However, these parameters are not mandatory for calculating IOL power in any of the formulas used. The biometry device utilized to obtain the biometric variables in our study, the IOL Master 500, is based on Partial-Coherence Interferometry (PCI), and is unable to measure CCT or LT values. Many surgeons worldwide still lack the opportunity to measure these parameters, as they use older biometers. Additionally, in certain cases, depending on the eye’s characteristics, newer biometers may not provide reliable LT values.

Among the strengths of our study, in addition to the strict inclusion and exclusion criteria, is the large number of formulas compared, including both widely used traditional formulas and newer generation formulas.

## Conclusions

In conclusion, our study reflects a superior accuracy of the Holladay 1_modified W-K_ formula, along with the Kane, Barrett, EVO and Pearl-DGS formulas in predicting refractive outcomes for eyes with an AL greater than 26 mm undergoing cataract phacoemulsification surgery. Statistically significant differences were observed when compared to other formulas, except for the Pearl-DGS formula.

## Electronic supplementary material

Below is the link to the electronic supplementary material.

Supplementary file1 (PDF 241 KB)

## Data Availability

Data is provided within the manuscript and supplementary information files. Additional data that support the findings of this study are available form the corresponding author, MV, upon reasonable request.

## Appendix

A. Hill W. Hill-RBF Calculator Version 3.0https://rbfcalculator.com/. Accessed 2 May 2024
B. Kane JX. Kane formula. https://www.iolformula.com/. Accessed 2 May 2024.
C. Yeo TK. EVO formula. https://www.evoiolcalculator.com/. Accessed 2 May 2024.
D. Barrett GD. Barrett Universal II formula. Asia-Pacific Association of Cataract and Refractive Surgeons. https://calc.apacrs.org/barrett_universal2105/. Accessed 2 May 2024.
E. Debellemanière G, Gatinel D, Saad A. Pearl-DGS formula. https://news.iolsolver.com/. Accessed 2 May 2024.
F. IOLCon. Steinbeis Vision Research. https://iolcon.org/. Accessed 2 April 2024.
