# Supplementary material for: Accuracy of 12 IOL power calculation formulas in highly myopic eyes
Source: Int Ophthalmol. 2025 Jun 25;45(1):264. doi: 10.1007/s10792-025-03608-0 (PMC12198256; doi:10.1007/s10792-025-03608-0)
Supplement: Supplementary file 1 — Supplementary file1 (PDF 241 KB) [file 10792_2025_3608_MOESM1_ESM.pdf]

Article Title: Accuracy of 12 IOL power calculation formulas in highly myopic eyes

Journal name: International Ophthalmology

Author names: Magí Vilaltella, Pau Cid-Bertomeu, Tonet Serés-Noriega, Valentín Huerva

Affiliation and e-mail address of the corresponding author: Department of Medicine and Surgery, Faculty of Medicine, University of Lleida, Spain. magi.vilaltella@udl.cat

Supplementary Table 1

Detailed results of One-sample t-Test comparing the ME (using ULIB constants) to zero

Formula	ME (SD), (D)	t-statistic	df	p-value	p-corrected (Bonferroni)
Hill RBF 3.0	0.153 (±0.450)	2.890	71	0.0051	0.061
Kane	0.180 (±0.429)	3.563	71	0.0007	0.0084
EVO	0.227 (±0.426)	4.510	71	<0.0001	<0.001
Barrett Universal II	0.176 (±0.405)	3.688	71	0.0004	0.0048
Pearl-DGS	0.135 (±0.427)	2.687	71	0.009	0.108
Holladay 2	0.477 (±0.502)	8.056	71	<0.0001	<0.001
Holladay 1	0.633 (±0.511)	10.510	71	<0.0001	<0.001
Haigis	0.308 (±0.473)	5.512	71	<0.0001	<0.001
SRK/T	0.321 (±0.524)	5.202	71	<0.0001	<0.001
Holladay $2_{NP\ W\text{-}K}$	-0.082 (±0.451)	-1.539	71	0.12	1.53

Holladay $1_{modified\ W\text{-}K}$	0.086 (±0.428)	1.709	71	0.091	1.10
SRK/$T_{modified\ W\text{-}K}$	0.079 (±0.475)	1.408	71	0.163	1.96

ME=mean error; SD=standard deviation; df=degrees of freedom

Supplementary Table 2

Detailed results of the Cochrane Q test and significant results of McNemar's chi-square tests comparing the percentage of hyperopic outcomes between formulas using ULIB constants

Formula		Q	Df	p-value	p-corrected (Bonferroni)
All formulas		139.88	11	<0.001	N/A
Formula comparison	% of hyperopic results	McNemar's chi-square (χ^2)	Df	p-value	p-corrected (Bonferroni)
Hill RBF 3.0 vs Holladay 1	67 vs 92	18	1	<0.0001	<0.001
Hill RBF 3.0 vs Holladay 2	67 vs 85	13	1	0.0002	0.013
Hill RBF 3.0 vs Holladay $2_{NP\ W\text{-}K}$	67 vs 43	15.21	1	0.0001	0.0066
BU II vs Holladay 1	71 vs 92	15	1	0.0001	0.0066
BU II vs Holladay $2_{NP\ W\text{-}K}$	71 vs 43	16.67	1	<0.0001	<0.001
Kane vs Holladay 1	71 vs 92	15	1	0.0001	0.0066
Kane vs Holladay $2_{NP\ W\text{-}K}$	71 vs 43	20	1	<0.0001	<0.001
EVO vs Holladay 1	73 vs 91	13	1	0.0002	0.013

EVO vs Holladay $2_{NP\ W\text{-}K}$	73 vs 43	22	1	<0.0001	<0.001
Pearl-DGS vs Holladay 1	68 vs 91	17	1	<0.0001	<0.001
Pearl-DGS vs Holladay 2	68 vs 84	12	1	0.0005	0.033
Pearl-DGS vs Holladay $2_{NP\ W\text{-}K}$	68 vs 43	18	1	<0.0001	<0.001
SRK/T vs Holladay $2_{NP\ W\text{-}K}$	76 vs 43	22.15	1	<0.0001	<0.001
Haigis vs Holladay $2_{NP\ W\text{-}K}$	76 vs 43	22.15	1	<0.0001	<0.001
Holladay 1 vs SRK/$T_{modified\ W\text{-}K}$	91 vs 65	19	1	<0.0001	<0.001
Holladay 1 vs Holladay $1_{modified\ W\text{-}K}$	91 vs 63	20	1	<0.0001	<0.001
Holladay 1 vs Holladay $2_{NP\ W\text{-}K}$	91 vs 43	35	1	<0.0001	<0.001
Holladay 2 vs SRK/$T_{modified\ W\text{-}K}$	84 vs 65	12.25	1	0.0005	0.033
Holladay 2 vs Holladay $1_{modified\ W\text{-}K}$	84 vs 63	15	1	0.0001	0.0066
Holladay 2 vs Holladay $2_{NP\ W\text{-}K}$	84 vs 43	30	1	<0.0001	<0.001
SRK/T vs Holladay $2_{NP\ W\text{-}K}$	76 vs 43	12.80	1	0.0004	0.026

Holladay 1$_{modified\ W\text{-}K}$ vs Holladay 2$_{NP\ W\text{-}K}$	63 vs 43	13.24	1	0.0003	0.019

BU II=Barrett Universal II; degrees of freedom

Supplementary Table 3

Significant results of the Wald test performed to elaborate the GEE

Formula	Wald χ^2	Df	p-value
Hill RBF 3.0	9.65	1	0.0019
Holladay 2	38.67	1	<0.001
Holladay 2$_{NP\ W\text{-}K}$	12.47	1	0.0004
Holladay 1	39.47	2	<0.001
Holladay 1$_{modified\ W\text{-}K}$	4.85	1	0.0277
SRK/T	44.41	2	<0.001
SRK/T$_{modified\ W\text{-}K}$	16.65	1	<0.001
BU II	5.44	1	0.0197
EVO	4.71	1	0.03
Kane	7.93	1	0.005
Haigis	12.78	1	0.0003

Df= degrees of freedom

Supplementary Table 4

Detailed results of the Friedman test and significant results of the Wilcoxon post-hoc test comparing absolute prediction errors between formulas after eliminating the systematic error

Formula	Friedman	Df	p-value	p-corrected (Bonferroni)
All formulas	618.07	11	<0.001	<0.001
**Comparison**	**MedAE**	**z-value (Wilcoxon)**	**p-value**	**p-corrected (Bonferroni)**
BU II vs SRK/T	0.246 vs 0.291	-3.376	0.0006	0.04
Kane vs SRK/T	0.233 vs 0.291	-3.373	0.0006	0.04
EVO vs SRK/T	0.252 vs 0.291	-3.578	0.0003	0.020
Holladay $1_{\text{modified W-K}}$ vs SRK/$T_{\text{modified W-K}}$	0.215 vs 0.3	3.606	0.0002	0.0132

MedAE=median absolute error; BU II=Barrett Universal II
